# Detection of Organophosphorous Chemical Agents with CuO-Nanorod-Modified Microcantilevers

**DOI:** 10.3390/s20041061

**Published:** 2020-02-15

**Authors:** Laurent Schlur, Pierre Agostini, Guillaume Thomas, Geoffrey Gerer, Jacques Grau, Denis Spitzer

**Affiliations:** 1 Nanomatériaux pour les Systèmes Sous Sollicitations Extrêmes (NS3E), UMR 3208 ISL/CNRS/UNISTRA, French-German Research Institute of Saint-Louis, 5, rue du Général Cassagnou, 68300 Saint-Louis, France; laurent.schlur@isl.eu (L.S.);; 2 Institut Jean Lamour, CNRS—Université de Lorraine, UMR 7198, Campus Artem, Allée André Guinier, 54011 Nancy, France; 3 Institute of Chemistry and Processes for Energy Environment and Health (ICPEES), UMR 7515 CNRS-University of Strasbourg, 67087 Strasbourg, France; 4 French-German Research Institute of Saint-Louis, 5, rue du Général Cassagnou, 68300 Saint Louis, France

**Keywords:** microcantilevers, sensors, nanostructured sensors, CuO/Cu(OH)_2_ nanorods, DMMP detection, organophosphorous, selective detection

## Abstract

Microcantilevers are really promising sensitive sensors despite their small surface. In order to increase this surface and consequently their sensitivity, we nanostructured them with copper oxide (CuO) nanorods. The synthesis of the nanostructure consists of the oxidation of a copper layer deposited beforehand on the surface of the sample. The oxidation is performed in an alkaline solution containing a mixture of Na(OH) and (NH_4_)_2_S_2_O_8_. The synthesis procedure was first optimized on a silicon wafer, then transferred to optical cantilever-based sensors. This transfer requires specific synthesis modifications in order to cover all the cantilever with nanorods. A masking procedure was specially developed and the copper layer deposition was also optimized. These nanostructured cantilevers were engineered in order to detect vapors of organophosphorous chemical warfare agents (CWA). The nanostructured microcantilevers were exposed to various concentration of dimethyl methylphosphonate (DMMP) which is a well-known simulant of sarin (GB). The detection measurements showed that copper oxide is able to detect DMMP via hydrogen interactions. The results showed also that the increase of the microcantilever surface with the nanostructures improves the sensors efficiency. The evolution of the detection performances of the CuO nanostructured cantilevers with the DMMP concentration was also evaluated.

## 1. Introduction

Nowadays, the threat related to the use of organophosphorous chemical warfare agents increases continuously [1]. This is why the detection of these compounds has been the aim of an increasing literature over the last years. The organophosphorous chemical warfare agents can be detected with a lot of technologies like gas chromatography [2,3], ion-mobility spectrometry [4], molecular imprinted polymer [5], MEMS [6], and microcantilevers [7,8].

Among all of these technologies, microcantilevers are promising sensors. These cantilevers which are identical to those used for atomic force microscopy (AFM) are sensitive to temperature [9], humidity [10], and mass variations due to sorption of molecules on their surface. The mass change due to sorption of molecules can be measured by resonance frequency shift in dynamic mode [11]. They are not only able to detect chemical warfare agents [7,8], they are also sensitive to explosives [12,13], or volatile organic compounds (VOCs) [14,15,16]. 

However, the small active area of these sensors limits their sensitivity. In order to improve the sensitivity, the cantilever surface can be nanostructured with TiO_2_ nanotubes or nanorods [17,18], ZnO nanorods or nanotubes [19,20,21], carbon nanotubes [22,23], mesoporous silica [24,25]. The surface can also be nanostructured with nanowires arrays [26,27]. Such nanostructures improve the efficiency of the microcantilever used for the detection of organophosphorous chemical warfare agents, VOCs [24,25], and explosives (detection at ppt scale) [17,24]. 

Cupric oxide (CuO) nanostructures are of great interest due to numerous application potentials. Few attempts were made to use the base-centered monoclinic CuO [28,29,30,31,32] nanostructures to detect chemical warfare agents, VOCs, H_2_S, explosives, and glucose. Cupric oxide 1D nanowires/ nanotubes arrays can be synthesized with thermal treatments [33], hydrothermal syntheses [34], and wet chemical syntheses [35]. In a previous article, the authors developed a wet chemical synthesis allowing the growth of CuO 1D nanostructures on silicon wafers [36]. They proved that the length and the diameter of the CuO nanostructures can be modified by changing the reactants concentration. In another previous article, the authors transferred the CuO nanostructures synthesis to piezoresistive cantilevers and developed a procedure allowing the mass production of CuO nanostructured piezoresistive cantilevers [16]. This procedure consists of the nanostructuration of a 4” wafer followed by several etching steps, allowing the fabrication of 450 nanostructured piezoresistive cantilevers. The disadvantage of this procedure is that all microcantilevers are nanostructured with 1D nanostructures having the same morphology (length and diameter).

The approach developed here aims to detect organophosphorous agents combines the high sensitivity offered by microcantilevers with the enhancement of the surface area offered by the 1D nanostructures. In this paper, the wet chemical method allowing the growth of CuO 1D nanostructures developed previously on silicon wafer [36] was adapted to microcantilevers with optical readout. A new cantilever nanostructuration procedure without the previously cited drawback was developed. The objective of this work is (i) to nanostructure individual commercial microcantilevers with CuO 1D nanostructures and (ii) to show that CuO is a good candidate for Chemical Warfare Agents CWA detection. This procedure offers the possibility to modify the morphology (length and diameter) of the nanostructures on each cantilever without requiring large mass production like it was the case for the piezoresistive cantilevers in the authors’ previous article. In this work, we explain and describe the entire procedure that we developed to nanostructure individual cantilevers. Microcantilevers were exposed to different vapors concentrations of an often used organophosphorous chemical warfare agent simulant, dimethyl methylphosphonate (DMMP), in order to evaluate the sensitivity [3,37]. 

## 2. Materials and Method

### 2.1. Growth of CuO Nanorods on Cantilevers with Optical Readout

The authors previously described a synthesis allowing the growth of CuO nanostructures on silicon wafers [16,36]. The objective is to transfer this synthesis to microcantilevers with optical readout. The nanostructures growing process was not modified when the wafer was replaced by a tipless silicon cantilever (Tipless Force Modulation AFM cantilever, TL-FM cantilever), except for the fact that the cantilever was not cleaned before it was used. These cantilevers, purchased from NanoAndMore, have a length of 225 ± 10 µm, a width of 28 ± 7.5 µm, a thickness of 3.0 ± 1 µm, a force constant comprised between 0.5 and 9.5 N/m and a resonance frequency between 45 and 115 kHz.

Before the synthesis of CuO nanostructures, a thin titanium layer (30 nm) and a thicker copper layer (700 nm) were deposited on the cantilever surface. These two layers were either evaporated under low vacuum pressure or sputtered. The sputtering device used is an Auto 306 (HHV Ltd, Crawley, UK) and the evaporation was done with a homemade device. For both techniques, the deposition speed is fixed to 1.5 A/s. For the sputtering process, the titanium (purity 99.97%, purchased from HHV Ltd) deposition was performed with an argon pressure of 2.2 × 10^−2^ mbar and a power density of 5.48 W/cm². During the sputtering of copper (purity 99.99%, purchased from HHV Ltd), the argon pressure and the power density were 2.2 × 10^−3^ mbar and 0.66 W/cm² respectively. For the evaporation under vacuum, the chamber pressure during the evaporation was close to 1.0 × 10^−6^ mbar. The titanium (purity: 99.9%) and copper (purity: 99.99%) sources were purchased from Umicore (Brussels, Belgium) and Unaxis (Pfäffikon, Swiss), respectively.

The next step consists of the growth of copper (II) hydroxide (Cu(OH)_2_) nanorods. Two reactants were used: sodium hydroxide (purity: 97%) and ammonium persulfate (purity: 98%) purchased from Honeywell and Sigma-Aldrich, respectively. To perform the synthesis, the cantilever is immerged for 15 min in a beaker at a distance from the bottom of 0.5 mm. The beaker was filled with an aqueous solution composed of 8 mL of NaOH (0.3125 mol/L), 4 mL of (NH_4_)_2_S_2_O_8_ (0.0313 mol/L) and 18 mL of distilled water. Then, the cantilever was immerged for 15 min in a second beaker containing an aqueous solution of 8 mL of NaOH (10 mol/L), 4 mL of (NH_4_)_2_S_2_O_8_ (1 mol/L) and 18 mL of distilled water. Here, the distance from the bottom was fixed to 5 mm.

In order to dehydrate copper (II) hydroxide into copper oxide (CuO), the cantilever covered with the previously grown Cu(OH)_2_ nanostructures was placed in a muffle furnace. Subsequently, the wafer was heated to 200 °C under static air with a heating rate of 3 °C/min. The cantilever was kept at 200 °C for 1 h before it was allowed to cool naturally down to room temperature.

The morphology and the size of the nanostructures were studied by Scanning Electron Microscopy (SEM), using a FEI (Hillsboro, USA) Nova NanoSEM 450 equipped with a Field Emission Gun. The cantilevers were also observed with a LEICA (Wetzlar, Germany) DM 2500M optical microscope.

### 2.2. Dimethyl Methylphosphonate Detection

**Figure 1:**
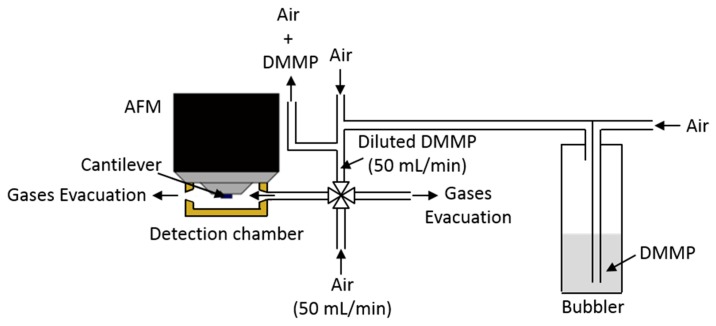
Scheme of the detection system.

The detection measurements were not performed directly with an organophosphorous chemical warfare agent but with a simulant. Dimethyl methylphosphonate (DMMP, purity 97%, purchased from Sigma-Aldrich) was used as a simulant. The detection measurements were done in a homemade detection system shown on Figure 1.

The detections tests were realized with an AFM PicoSPM LE^®^ in a detection chamber with a volume of 20.047 cm^3^. The cantilever is centered inside the detection chamber. Pure air or air containing DMMP can be injected in this chamber. The DMMP vapors were generated with an air flow going through a bubbler containing DMMP. The DMMP vapor concentration can be modified by using a dilution stage. 

Before the stabilization of the cantilever resonance frequency, 50 mL/min of pure air was injected inside the cantilever chamber. After this stabilization step, 50 mL/min of air containing DMMP was injected inside the detection chamber for 10 minutes. Finally, 50 mL/min of pure air is injected again in the cantilever chamber in order to desorb the DMMP from the cantilever surface. 

The air used for the experiments was purchased from AirLiquid^®^ (Alphagaz^TM^ 2) and contains less than 500 ppb of water. For the detection measurements, the bubbler temperature was fixed to 19 °C and the other parts of the detection system were maintained at a temperature of 22 °C. The DMMP concentration present in the detection chamber was determined in real time by gas chromatography-mass spectrometry (GC-MS). The analysis was carried out using an Agilent GC–MS system consisting of a 7890A GC and a 5973 mass detector. An Agilent DB-5 (5 m × 0.1 mm i.d. × 0.15 µm) GC column was used. The vapor sample was injected in the split mode using helium as carrier gas at a constant flow of 0.5 mL/min and pressure of 1.8 bar. The column temperature was fixed at 60 °C. The MS source and MS quad temperature were programmed at 230 °C and 150 °C, respectively. MS data were acquired using ChemStation (version E.02.02.1431).

All the detection measurements were repeated at least three times. The detection curves representing the evolution of the resonance frequency versus the measurement time were not post-treated, except for the baseline in some cases. Indeed, the baseline was flattened when there was a small drift of the microcantilever resonance frequency. 

## 3. Results and Discussion

### 3.1. Growth of CuO Nanorods on the Surface of a Cantilever with Optical Readout

**Figure 2:**
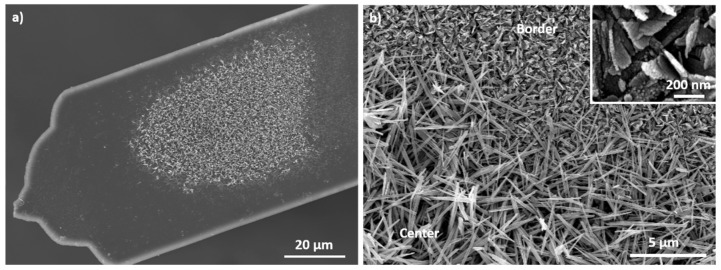
SEM images of CuO nanostructures grown on the surface of AFM cantilever. (**a**) Overview of the sensor. (**b**) Cantilever border (Insert: high magnification on the nanostructures present on the border).

The nanorods were synthesized on the cantilever surface as described previously. Figure 2 shows the cantilever surface after the growth of CuO nanostructures. 

**Figure 3:**
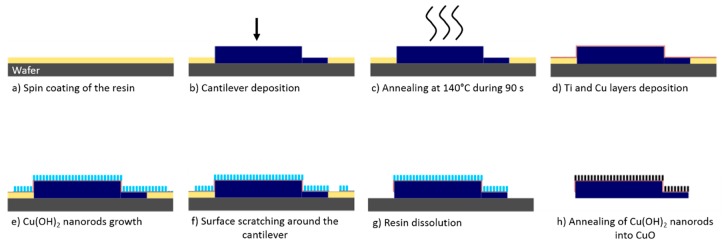
Schematic representation of the protocol developed to overcome the border effect observed during the nanorods synthesis on the cantilever surface.

Nanorods are only present at in the center of the microcantilevers. The border is covered with nanosheets (Figure 2b). The authors showed in a previous article that the nanorods and the nanosheets are cupric oxide and copper hydroxide respectively [36]. They proved also that, an increase of the chemical reaction time leads to the transformation of Cu(OH)_2_ nanorods into CuO nanosheets because copper hydroxide is unstable in alkaline solutions [36]. At the border of the sample, this transformation occurs faster than in the center. This phenomenon can be explained by the close distance between the wafer and the beaker bottom during the first reaction, hindering the reactant renewal in the center of the sample and consequently slowing down the dissolution of copper hydroxide. Even a decrease of both reaction times to one or two minutes do not prevent this border effect. So, the formation of CuO nanosheets on the border occurs during the first seconds of the reaction. Several other modifications of the synthesis (reactant concentrations, temperature, and reactant purity) were tested in order to avoid this border effect without success. To overcome this border effect, the synthesis protocol was consequently modified. The new protocol is given on Figure 3 and described in the following paragraph. 

A wafer (1.5 × 1.5 cm², one side polished, purchased from Siegert Consulting e.K) was successively cleaned in an acetone and ethanol solution before being dried under a N_2_ atmosphere. The wafer was exposed to an oxygen plasma treatment for 10 min in order to eliminate last organic traces. A layer of resin (AR-PC 504 purchased by Allresist GmbH) was spin coated on the wafer surface (Figure 3a). Immediately after this step and before the evaporation of the solvent present in the resin, the cantilever was delicately and manually deposited on the resin surface with tweezers (Figure 3b). In order to remove the resin present between the cantilever and the wafer, tweezers were used to press top down the sensor. The movement path of the sensor is symbolized by the arrow visible on Figure 3b. The objective is to avoid discontinuities between the cantilever surface and the resin surface (i.e., the cantilever thickness and the resin thickness are identical) in order to avoid the border effect. In this case, the cantilever and the resin surfaces constitute one single surface with no discontinuity as the cantilever is surrounded by a resin having exactly the same thickness. Consequently, the border effect will not appear anymore at the limits of the cantilever, but it will occur at the edges of the resin surface. In order to have the cantilever and resin surfaces at the same height, the resin was spin coated for 10 seconds at different rotation speeds: 1000 rpm, 2000 rpm, and 2250 rpm. The acceleration was fixed to 1000 rpm/s. After the cantilever deposition, the wafer was heated on a hot plate at 140 °C for 90 seconds in order to obtain a hard-baked film (Figure 3c). Then, the titanium and the copper layers were deposited (Figure 3d) and the Cu(OH)_2_ nanorods were synthesized (Figure 3e) as described previously. The nanorods grow on the cantilever surface and also on the resin surface with no discontinuity. Next, the surface around the cantilever was scratched under binocular with a needle fixed on a micromanipulator in order to detach the nanorods film present on the cantilever surface from the nanorods film fixed on the resin surface (Figure 3f). After that, the resin was dissolved successively in several acetone solutions (Figure 3g) and the cantilever was dried under N_2_ flow. Finally, the Cu(OH)_2_ nanorods were dehydrated into CuO as explained previously (Figure 3h).

This developed procedure prevents also all possible growth of the nanorods on both sides of the cantilevers because one side is confined in the resin. A growth of the nanorods on both sides of the cantilever would prevent the reflection of the laser leading to the impossibility to measure the resonance frequency.

**Figure 4:**
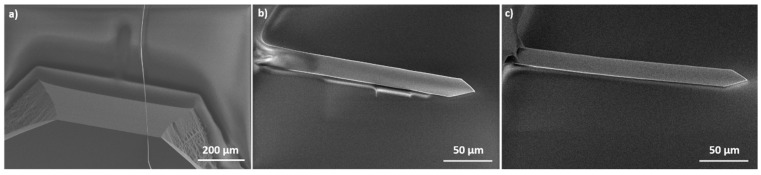
SEM (**a**) top view and (**b**,**c**) titled view of cantilevers deposited in the resin. The resin was spin coated at different rotation speeds: (a) 1000 rpm, (b) 2000 rpm and (c) 2250 rpm.

As explained previously, the surface of the resin has to be at the same height than the cantilever surface. To obtain such a result, different spin coating speeds were applied. Samples were observed by SEM (Figure 4). 

For a rotation speed of 1000 rpm (Figure 4a), the cantilever is completely immerged in the resin and for a value of 2000 rpm (Figure 4b) the cantilever is still partially covered. However, for a rotation speed of 2250 rpm, the resin does not cover anymore the cantilever. Indeed, the resin is at the same height than the sensor surface. The manufacturer’s data indicate that for a rotation speed of 2250 rpm, the resin thickness is equal to 2.8 µm. This value is nearly equal to the theoretical cantilever thickness which is equal to 3.0 µm ± 1.0 µm. Therefore, the spin coating rotation speed of the resin is fixed to 2250 rpm.

**Figure 5:**
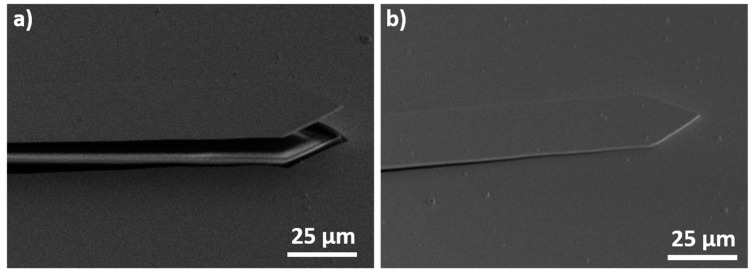
SEM tilted views of cantilevers in the resin after the deposition of the titanium/copper layers by (**a**) sputtering and (**b**) evaporation under vacuum.

The wafer is next heated on a hot plate at 140 °C for 90 s in order to obtain a hard-baked resin film. The following step of the protocol consists of depositing the titanium and copper layers. These two layers were obtained either by sputtering or by evaporation under vacuum. Figure 5 shows the cantilevers in the resin after the titanium/copper layers deposition.

Figure 5 shows that after the sputtering process the cantilever is outside of the resin, which is not the case after the evaporation under vacuum. After the evaporation process, the microcantilever and the resign have the same height. The modification of the cantilever position during the sputtering is due to the argon plasma which etches the resin during the first moments of the sputtering process. This etching releases the cantilever from the resin, so the cantilever surface is not anymore at the same height that the resin surface. In order to prevent this effect, the titanium and the copper layers are deposited by evaporation under vacuum in the rest of this article. 

**Figure 6:**
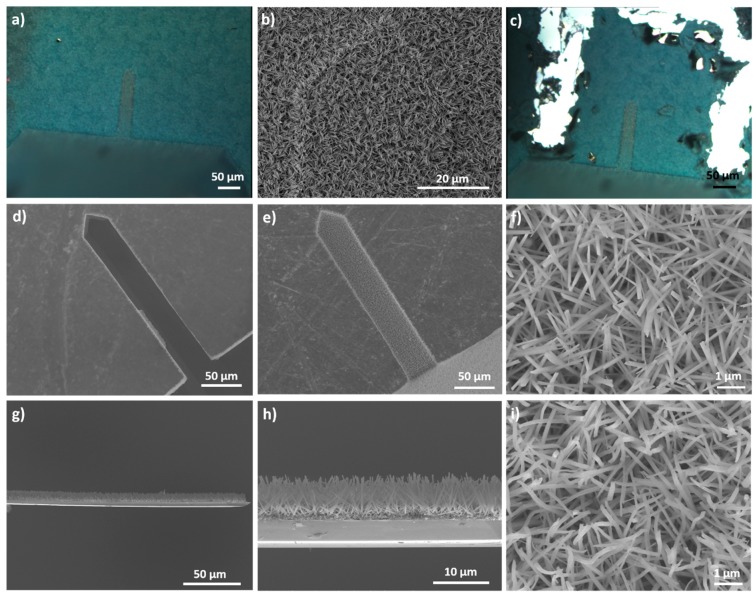
(**a**) Optical microscope image and (**b**) SEM top view of Cu(OH)_2_ nanorods grown on the cantilever just after the synthesis. (**c**) Optical microscope image of the sample after scratching around the cantilever surface with a needle fixed on a micromanipulator. (**d**–**f**) SEM top views of the cantilever. (**d**) Cantilever side which used for the laser reflection (which was in the resin). (**e**,**f**) Cantilever side covered with the nanorods. (**g**,**h**) SEM side view of the nanostructured cantilever. (**i**) SEM top view of the cantilever side with the nanorods after the annealing at 200 °C for 1 h.

The results of the next synthesis steps are presented on Figure 6.

The next step is to grow copper hydroxide nanorods. Figure 6a shows that the entire surface of the sample is blue after the synthesis and that there is no discontinuity between the cantilever and the resin surface. Figure 6b confirms this result. This Figure proves also that nanorods are present on the cantilever surface. All the cantilever surface is homogeneous, which proves that there is no border effect. The authors proved in a previous article with X-ray diffraction analyses that this blue color is attributed to copper hydroxide [36]. 

After the Cu(OH)_2_ synthesis, in order to separate the nanorods on the cantilever from the ones on the film, the surface surrounding the cantilever was mechanically scratched. This was performed using a needle attached to a micromanipulator under a microscope (Figure 6c). This step is essential to avoid the breaking of the cantilever during the resin dissolution. Indeed, without this step, the nanorods film present on the resin surface would keep attached to the cantilever which could break easily under the mass of this film during the resin dissolution.

Figure 6d–f shows the two surfaces of the cantilever after the resin dissolution. One of the two surfaces, is homogeneously covered with nanorods having a diameter of 114 ± 24 nm (Figure 6e–f). This result confirms that there is no border effect and that the nanorods grow on the entire surface of the cantilever. The nanorods density is approximately 1.4 × 10^9^ nanorods/cm^2^, corresponding to ca. 90,000 nanorods per microcantilever. Despite the fact that the authors used the same chemical conditions (reaction time and reactants concentration), as in the previous work [36], Cu(OH)_2_ nanorods were obtained instead of Cu(OH)_2_ nanotubes. This difference can be explained by the fact that we did not use the same NaOH powder in the both articles. Indeed, by using the NaOH powder used in the previous article, the authors achieved to obtain nanotubes with the protocol developed in this article. This result proves that the impurities present in the NaOH powder influence the morphology of the nanostructures.

The other side of the cantilever (which was protected by the resin) is not covered with nanorods (Figure 6d) and can consequently reflect the laser used to measure the resonance frequency. We also observed that a very small part on the edge of this side stills remain covered by nanorods. As we applied a mechanical and hand pressure of the microcantilever during deposition in the resin, this small part was not directly in contact with the resin, leading to the growth of nanorods during the synthesis.

The side views (Figure 6g–h) of the cantilevers confirm that the nanorods grow only on one side of the cantilever. The Cu(OH)_2_ nanorods are oriented rather perpendicularly to the cantilever surface and they have a length of 6.98 ± 0.66 µm. The presence of the Cu(OH)_2_ nanorods on the cantilever surface increases the surface area by a factor of 35 compared to the surface of the raw cantilevers.

The last step of the protocol consists of annealing of the copper hydroxide nanorods at 200 °C (Figure 6i). After annealing, nanorods are still present on the cantilever surface. The authors proved in a previous publication that this annealing allows the dehydration of copper (II) hydroxide into copper (II) oxide [36]. The nanorods have a diameter of 125 ± 30 nm. The morphology of the nanorods before and after annealing is almost identical. The annealed rods look less straight than the non-annealed ones. This is due to a change of the crystallographic structure.

Consequently, it is possible to homogeneously nanostructure all the cantilever surfaces with Cu(OH)_2_ or CuO nanorods by following the developed synthesis protocol. These cantilevers can now be used for the detection of organophosphorous agents.

### 3.2. Detections Measurements

To detect DMMP, the resonance frequency (*f_r_*) of the cantilever, which is a harmonic oscillator [38], is continuously measured. After the adsorption of DMMP onto the cantilever surface, the resonance frequency (*f_r_*) decreases because *f_r_* depends on the cantilever mass [10], according to (1)

(1) $$f_{r}=\frac{1}{2\pi }\sqrt{\frac{k}{m^{*}}}$$

where *k* is the spring constant and m* is the effective mass of the cantilever. The effective mass is equal to nm_b_, where m_b_ is the cantilever beam mass and n is a geometrical factor depending on the shape of the cantilever equal to 0.24 for a rectangular cantilever [10]. When DMMP is adsorbed on the cantilever surface, the mass increases, and consequently the resonance frequency decreases. 

The performance of the CuO nanostructured cantilevers previously synthesized were tested and compared with the performance of a raw TL-FM cantilever and with a cantilever covered with a CuO layer. This CuO layer was obtained by annealing at 350 °C during one hour under air, a copper layer previously evaporated on the cantilever surface (see elaboration details in the Supplementary Materials, Figure S1). XPS analyses confirm that after annealing, the surface of the layer in contact with DMMP is essentially composed of cupric oxide (CuO). The XPS results are presented in detail in the Supplementary Materials (Figure S2). Figure S3 shows the morphology of the CuO layer. This layer is characterized by a slight roughness resulting from the formation of nanoparticles clusters. The CuO layer is rougher than the raw TL-FM cantilever which has a really smooth surface (see Figure S3).

**Figure 7:**
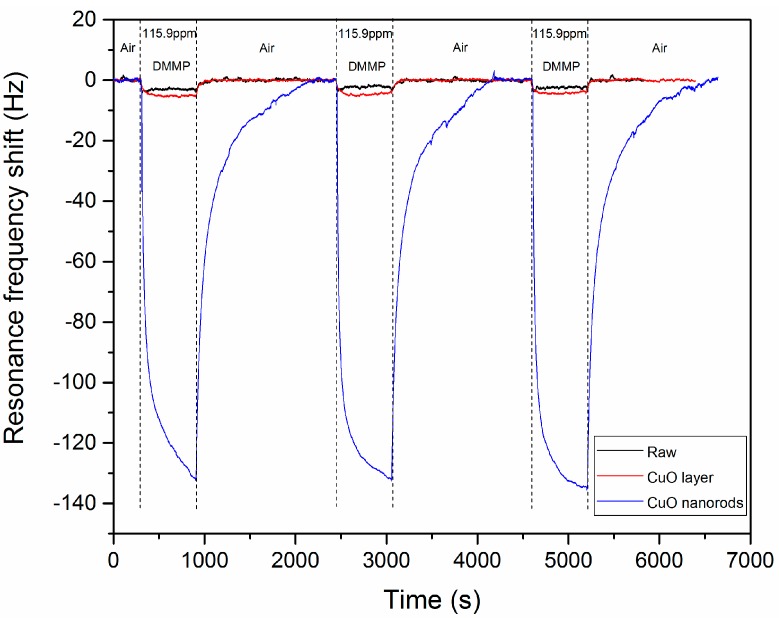
Resonance frequency shift of a raw cantilever (black), one covered with a CuO layer (red) and one nanostructured with CuO nanorods (blue). The cantilevers were exposed three times for 10 min to 115.9 ppm of DMMP. After each DMMP exposure, pure air is injected in the chamber containing the cantilever.

The raw cantilever, the cantilever covered with a CuO layer and the CuO nanostructured cantilevers were exposed for 10 min to 115.9 ± 0.9 ppm of DMMP. The detection results are illustrated in Figure 7.

A fast decrease of the resonance frequency when DMMP vapors are put into contact with microcantilevers is observed. This resonance frequency shift of 132.9 ± 1.6 Hz is due to the adsorption of DMMP on the CuO nanorods surface. This adsorption occurs thanks to the formation of hydrogen bonds between the hydroxyl groups present on the CuO surface and the P=O function of DMMP. So, the CuO nanostructured cantilevers are sensitive to DMMP vapor. After 10 min, the DMMP vapors in contact with the cantilever are replaced with pure air without DMMP. The resonance frequency of the nanostructured cantilever returns to its baseline value, which means that all the DMMP desorbs from the CuO nanorods surface. This complete desorption is due to the weak interactions of the hydrogen bonds formed between DMMP and the hydroxyl function of CuO. Moreover, after three cycles of adsorption/desorption, the sensitivity of the cantilever is not modified (Figure 7). The weak interactions guarantee a good regeneration of the microcantilever and a good repeatability. In a previous work, we showed that CuO was not able to detect the tested explosives (PETN, RDX) and VOCs (toluene, benzene, trichloroethylene, tetrachloroethylene, xylene-p, acetaldehyde, and acrolein) [16]. This work here proves that CuO can still be used as a sensing material for simulant of organophosphorous compounds, because it detects at least DMMP.

The much lower resonance frequency shift obtained with the cantilever covered with a CuO layer (4.9 ± 0.5 Hz), compared to the nanostructured cantilever proves that the nanostructures improve significantly the detection performances. This is owing to an increase of the sensor surface, which confirms results previously published by the authors [17]. For the raw silicon cantilever, the resonance frequency shift is 2.5 ± 0.5 Hz. This value is slightly lower than the shift observed for the cantilever covered with a CuO layer, which can be due to the difference of roughness observed between the two types of sensors (see Figure S3). Indeed, the CuO layer is rougher, so the cantilever surface which can interact with DMMP is higher. The difference of detection response can also be explained by the fact that CuO and the native silica layer present on the raw cantilever surface may not have the same affinity toward DMMP.

**Figure 8:**
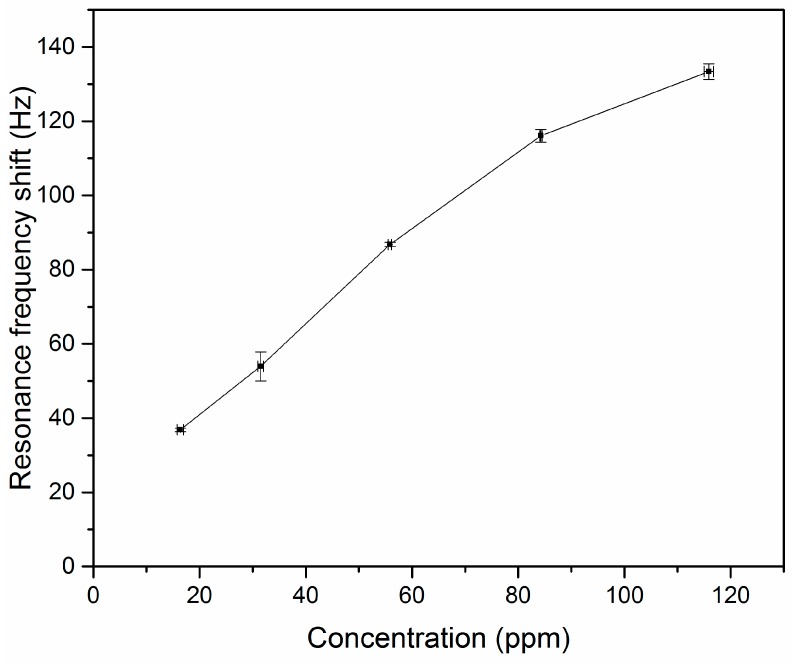
Evolution of the resonance frequency shift of the CuO nanostructured cantilever exposed to several DMMP concentrations. The tested concentrations are: 115.9 ppm, 84.3 ppm, 55.9 ppm, 31.5 ppm, and 16.4 ppm.

In order to determine the lowest DMMP concentration which can be detected by the CuO nanostructured cantilever, the DMMP concentration in contact with the cantilever has been modified. The tested DMMP concentrations are: 115.9 ppm, 84.3 ppm, 55.9 ppm, 31.5 ppm, and 16.4 ppm. The evolution of the absolute value of the resonance frequency shift of the CuO nanostructured cantilever as function of the DMMP concentration is visible on Figure 8. 

The CuO nanostructured cantilever is able to detect all tested DMMP concentrations (115.9 ppm, 84.3 ppm, 55.9 ppm, 31.5 ppm, and 16.4 ppm). For the lowest tested DMMP concentration (16.4 ppm), the resonance frequency shift is 36.8 ± 0.5 Hz. Clearly, the cantilever can detect lower DMMP concentrations, but the GC-MS device used here to control the DMMP concentrations cannot measure lower concentrations. The shape of the curve (Figure 8) does not allow direct extrapolation of the evolution of the resonance frequency shift for lower concentrations than 16.4 ppm. Consequently, the lowest DMMP concentration which can be measured with the CuO nanostructured cantilevers is below 16.4 ppm.

## 4. Conclusions

In summary, we nanostructured commercial cantilevers with an optical readout using cupric oxide nanorods. These nanorods have a diameter of 125 nm and a length close to 7 µm. The synthesis consists of oxidizing a homogeneous copper layer, previously evaporated on the cantilever surface. The oxidation reaction is performed in an alkaline aqueous solution containing Na(OH) and (NH_4_)_2_S_2_O_8_. The synthesis, which was developed on a silicon wafer, could not be directly transferred to a cantilever due to a border effect. This effect occurred during the synthesis, preventing nanorod growth on the entire cantilever surface. The development of a specific masking procedure and the optimization of the copper layer deposition were necessary to overcome this border effect and so to synthesize CuO nanorods on all the cantilever surface.

The CuO nanostructured cantilevers were tested as sensors for organophosphorous chemical warfare agents. DMMP vapor was used as a simulant for the detection tests. The CuO nanostructured cantilevers are able to detect DMMP. They are 27 times more sensitive than the cantilevers covered with a layer of CuO when they are exposed to 115.9 ppm of DMMP. Therefore, the increase of the surface area of the cantilever by a factor of 35 significantly improves its sensing performance. Moreover, copper oxide reveals to be a very good sensing material for organophosphorous compounds as it detects DMMP but not other substances like explosives (PETN and RDX) and some VOCs such as toluene, benzene, trichloroethylene, tetrachloroethylene, xylene-p, acetaldehyde, and acrolein. The lowest DMMP concentration which was detected in this article by the CuO nanostructured cantilevers is 16.4 ppm. For technical reasons, lower DMMP concentrations could not be tested, but the developed sensor can clearly detect smaller DMMP amounts.

Our work will now focus at improving the DMMP vapor generation system in order to be able to measure lower DMMP concentrations. Another objective is to optimize the sensors surface by changing the length and diameter of the nanorods in order to improve the microcantilever sensitivity.

## Supplementary Materials

The following are available online at https://www.mdpi.com/1424-8220/20/4/1061/s1, Figure S1: Schematic representation of the protocol used in order to deposit a dense CuO layer on the surface of the cantilever, Figure S2: O1s and Cu2p3/2 XPS spectra of the copper layer annealed at 350 °C during one hour under air, Figure S3: SEM top view of a) a raw TL-FM cantilever and b) the same cantilever covered with a CuO layer. The particles visible on picture a) are dust particles present on the cantilever and which were used to adjust the focus on the cantilever surface.
