# Supplementary material for: Detection of Organophosphorous Chemical Agents with CuO-Nanorod-Modified Microcantilevers
Source: Sensors (Basel). 2020 Feb 15;20(4):1061. doi: 10.3390/s20041061 (PMC7070513; doi:10.3390/s20041061)
Supplement: Supplementary file 1 [file sensors-20-01061-s001.pdf]

sensors

MDPI

Article

Detection of Organophosphorous Chemical Agents with CuO Nanorods modified Microcantilevers

Laurent Schlur [1,†], Pierre Agostini [1,2,†], Guillaume Thomas [1], Geoffrey Gerer [1,3], Jacques Grau [4], and Denis Spitzer [1,†,*]

[1] Nanomatériaux pour les Systèmes Sous Sollicitations Extrêmes (NS3E), UMR 3208 ISL/CNRS/UNISTRA, French-German Research Institute of Saint-Louis, 5, rue du Général Cassagnou, 68300 Saint-Louis, France; laurent.schlur@isl.eu (L.S.); pierre.agostini@univ-lorraine.fr (P.A.); guillaume.thomas@isl.eu (G.T.); geoffreyge@hotmail.com (G.G.)

[2] Institut Jean Lamour, CNRS - Université de Lorraine, UMR 7198, Campus Artem, Allée André Guinier, 54011 Nancy, France

[3] Institute of Chemistry and Processes for Energy Environment and Health (ICPEES), UMR 7515 CNRS-University of Strasbourg, 67087 Strasbourg, France

[4] French-German Research Institute of Saint-Louis, 5, rue du Général Cassagnou, 68300 Saint Louis, France; jacques.grau@isl.eu

* Correspondence: denis.spitzer@isl.eu; Tel.: +33-389695075

† These authors contributed equally to this work.

Received: 24 January 2020; Accepted: 13 February 2020; Published: 15 February 2020

Elaboration of the cantilever covered with a CuO layer

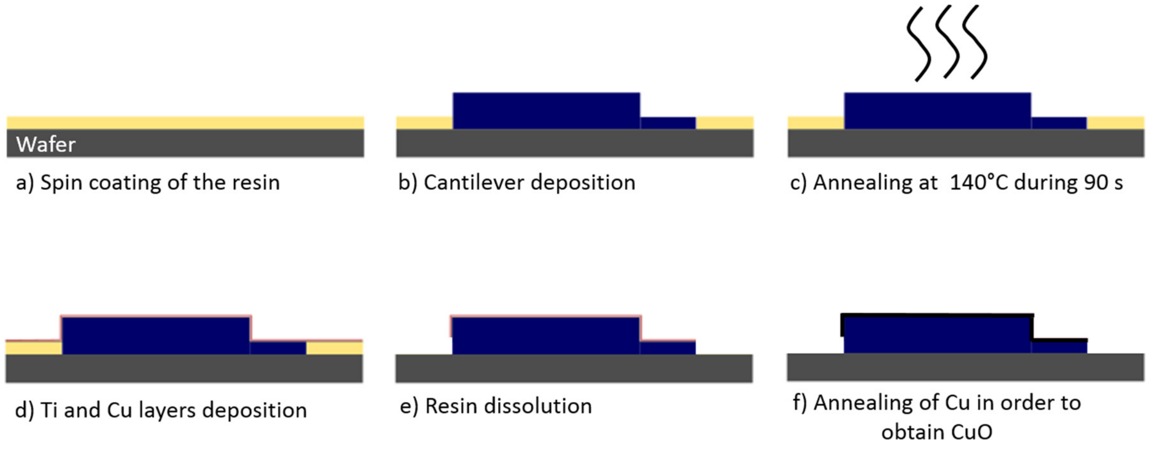

Figure S1. Schematic representation of the protocol used in order to deposit a dense CuO layer on the surface of the cantilever.

Protocol: A 1.5 × 1.5 cm^2 wafer was cleaned like explained previously. A layer of resin (AR-PC 504 purchased by Allresist GmbH) was spin coated on the wafer surface at a rotation speed of 2250 rpm (Figure S1a) with an acceleration speed of 1000 rpm/s. Immediately after this step and before the evaporation of the solvent present in the resin, the cantilever was deposited in the resin (Figure S1b). Then, the wafer was heated on a hot plate at 140 °C during 90 s (Figure S1c). After this annealing step, 30 nm of titanium and 700 nm of copper were evaporated (Figure S1d) on the cantilever surface with the same conditions than described previously in this article. The presence of resin avoids the deposition of copper on the other face of the cantilever. After that, the resin was dissolved in an acetone solution (Figure S1e). Finally the cantilever was heated to 350 °C, under static air with a heating rate of 3 °C/min (Figure S1f). The cantilever was kept at 350°C for 1 h before it was cooled down with a rate of 1 °C/min until room temperature. This heating step is used to oxidize the copper layer into a CuO layer.

XPS Analyzes of the Copper Layer Annealed at 350°C During One h under Air

X-ray Photoelectron Spectroscopy (XPS) is performed on a VG SCIENTA SES-2002 spectrometer equipped with a hemispherical energy analyser. An Al $K_{\alpha1,2}$ monochromatic X-ray source (1486.6 eV) is used. The spectra are acquired at a constant take-off angle of 90°. The high resolution scans are recorded using 100 eV pass energy, whereas the wide scans are recorded using 500 eV pass energy. The X-ray excitation source power is equal to 420 W (14 kV, 30 mA) and the vacuum pressure in the analysis chamber is close to 10^{-9} mbar. The spectrometer is calibrated using values of 84 eV, 368.2 eV and 932.7 eV for Au4f7/2, Ag3d5/2 and Cu2p3/2 respectively. All the spectra are referenced according to the C1s peak at 285.0eV. Deconvolution of the peaks is carried out using CasaXPS software (version 2.3.18dev1.1l) by using Gaussian-Lorentzien (70%–30%) fitting curves and full widths at half maximum which are quite equivalent within a same peak.

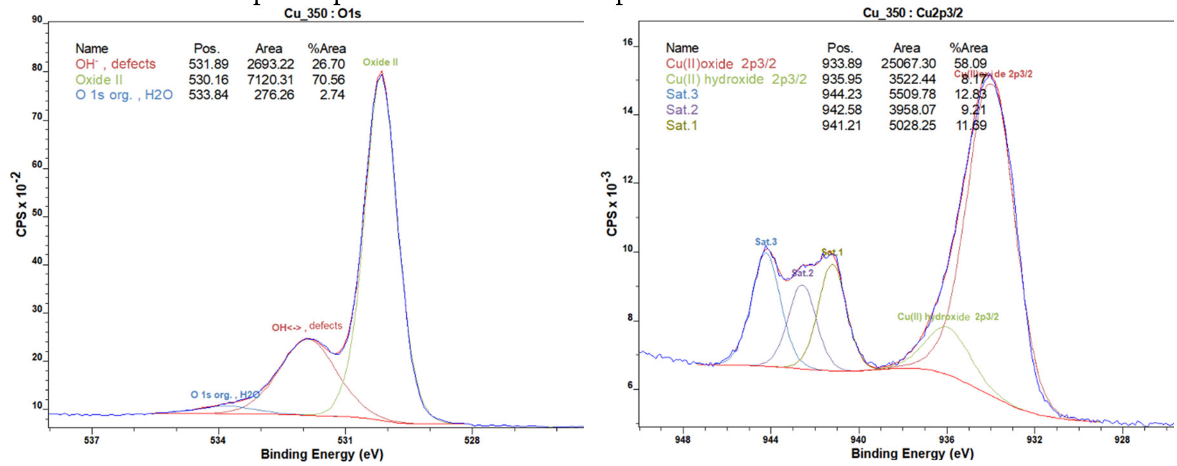

Figure S2. O1s and $Cu2p^{3/2}$ XPS spectra of the copper layer annealed at 350 °C during one hour under air.

Results: The O1s spectrum displayed in Figure S2 shows that the surface of the sample is mainly an oxide, but hydroxides with defects are also present. Some water traces and organic oxygen can also be detected.

The copper $Cu2p^{3/2}$ spectrum (Figure S2) is composed of satellites peaks (Sat.1-2-3) corresponding to paramagnetic Cu^{2+} copper. With copper II, the sum of the satellites areas represents normally the half of the $Cu2p^{3/2}$ peak area. As it is the case here, the presence of copper II is confirmed. The surface of the layer is predominantly composed of CuO (88 at%) and a small quantity of copper hydroxide (12 at%) which is present at the extreme surface.

SEM Pictures of the Cantilever Surface

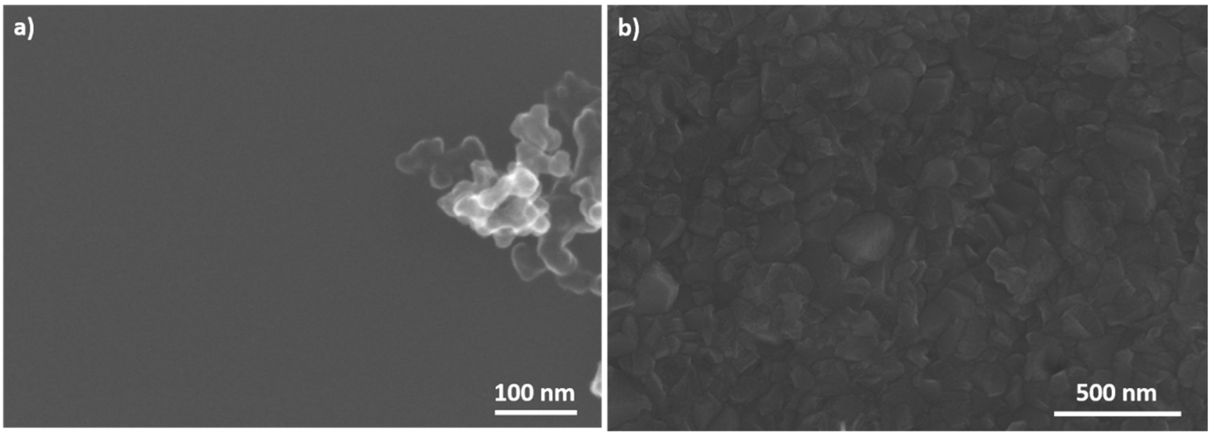

Figure S3. SEM top view of **a**) a raw TL-FM cantilever and **b**) the same cantilever covered with a CuO layer. The particles visible on picture **a**) are dust particles present on the cantilever and which were used to adjust the focus on the cantilever surface.
